# Cumulative endpoint and reconstructed 500-m energy-system contributions from individualized pacing-matched trials in 2000-m rowing ergometry

**DOI:** 10.3389/fphys.2026.1840466

**Published:** 2026-09-29

**Authors:** Jiashi Lin, Xiaoning Ma, Caoan Yuan, Lei Yi

**Affiliations:** 1 College of Physical Education, Jimei University, Xiamen, Fujian, China; 2 Xianyang Yucai Tianjiabing Middle School, Xianyang, Shaanxi, China

**Keywords:** cumulative endpoint, energy-system contribution, individualized pacing, pacing replication, reconstructed 500-m segment, rowing ergometer

## Abstract

**Objective:**

To evaluate the accuracy with which 500-, 1000-, and 1500-m endpoint trials reproduced each rower’s individualized 2000-m pacing template, estimate cumulative energy-system contributions at 500, 1000, 1500, and 2000 m, and reconstruct contributions across four successive 500-m segments.

**Methods:**

Twelve trained male provincial-team rowers completed a 2000-m all-out rowing-ergometer reference trial to establish individualized cumulative time, mean power, and mean stroke-rate targets, followed by pacing-matched 500-, 1000-, and 1500-m endpoint trials. Pacing-replication accuracy was evaluated using achieved-minus-target bias, MAE, MAPE, concordance, and Bland–Altman limits of agreement. Energy-system contributions were estimated indirectly using the PCr–La–O_2_ framework. Cumulative endpoints and within-rower reconstructed 500-m segments were analyzed separately using repeated-measures analyses with Holm-adjusted comparisons.

**Results:**

At 500, 1000, and 1500 m, time MAE was 0.050, 0.092, and 1.258 s; power MAE was 0.667, 0.917, and 5.083 W; and stroke-rate MAE was 0.750, 0.396, and 0.359 spm, respectively. Corresponding MAPE values were 0.053–0.431% for time, 0.157–1.339% for power, and 1.239–2.483% for stroke rate. No stable group-level systematic bias was detected after Holm adjustment, although statistical equivalence was not tested. Reconstruction showed a higher estimated phosphagen contribution during 0–500 m, glycolytic contribution concentrated mainly within the first 1000 m, and a higher aerobic contribution after the opening segment. No robust segmental difference in total estimated contribution was concluded.

**Conclusions:**

Individualized known-endpoint trials can closely reproduce a rower’s 2000-m pacing template while providing endpoint-specific post-exercise inputs for cumulative indirect energy-system estimation. Within-rower reconstruction offers a transparent way to describe successive 500-m energetic patterns alongside actual kinematics, but these reconstructed segments should not be interpreted as direct physiological measurements of continuous race phases or as a validated training prescription.

## 1 Introduction

Contemporary evaluation of 2000-m rowing-ergometer performance is multidimensional and cannot be reduced to a single energy-system percentage. A recent systematic review identified maximal oxygen uptake and peak power output as the variables most consistently associated with 2000-m ergometer performance, with lactate-threshold-related power and critical power also showing important associations (Borges et al., 2025). Classic and more recent rowing studies likewise indicate that aerobic power, threshold-related capacity, and maximal power represent complementary dimensions of rowing-performance assessment (Ingham et al., 2002; Turnes et al., 2020; Kilbey et al., 2025; Possamai et al., 2024). Recent sport-specific cardiopulmonary testing in international-level medal-winning boat-sport athletes, including male rowers, has further provided preliminary reference values for power output, peak lactatemia, and maximal oxygen uptake, reinforcing the complementary role of these measures in physiological profiling (Crisafulli et al., 2025b). Estimation of energy-system contribution addresses a narrower but complementary question: how oxidative and non-oxidative pathways jointly meet the demands of a high-power 2000-m task usually completed in approximately 5–8 min. Previous research generally supports aerobic predominance while indicating that phosphagen and glycolytic pathways remain important during changes in power and pacing (Hagerman, 1984; Pripstein et al., 1999; de Campos Mello et al., 2009; Kennedy and Bell, 2003; Brown et al., 2010).

A continuous 2000-m test can describe within-trial changes in power, stroke rate, heart rate, and oxygen uptake. However, pacing strategy may alter the time course of blood-lactate accumulation, perceived exertion, and oxygen-uptake-related responses, while shorter all-out distances may elicit power output and relative anaerobic demands that differ from those of a 2000-m effort (Boillet et al., 2022; Astridge et al., 2024). Therefore, separate shorter endpoint trials cannot be interpreted as direct physiological replicas of the same distances reached during one continuous 2000-m test. This distinction is particularly important for endpoint energy estimation, because values obtained after termination at 500, 1000, or 1500 m represent cumulative responses from independent trials performed on different days rather than direct observations of independent segments within a single race.

Previous studies have mainly estimated energy-system contributions across an entire 2000-m trial or compared independent maximal trials performed over different distances or durations (Pripstein et al., 1999; de Campos Mello et al., 2009; Li et al., 2017; Astridge et al., 2024). Li et al. (2017) used self-paced all-out trials of prescribed duration performed in random order. López-Sánchez et al. (2018) used shorter trials that reproduced split time and stroke rate from an initial 2000-m performance but did not estimate the three energy systems using the PCr–La–O_2_ framework. Yan (2025) reconstructed successive 500-m contributions using randomized unknown-endpoint termination trials. These designs provide complementary evidence but have not simultaneously combined formal verification of individualized known-endpoint pacing replication, cumulative endpoint estimation, and within-rower reconstruction of successive 500-m segments.

Despite these advances, it remains unclear how accurately shorter known-endpoint trials can reproduce each rower’s individualized 2000-m pacing template and how cumulative endpoint estimates obtained under this design can be used to reconstruct energetic patterns across successive 500-m segments. The present study therefore evaluated the pacing-replication accuracy of individualized 500-, 1000-, and 1500-m endpoint trials, estimated cumulative phosphagen, glycolytic, and aerobic contributions at 500, 1000, 1500, and 2000 m, and reconstructed successive 500-m contributions at the individual level. The resulting segmental patterns were also compared descriptively with those reported using Yan’s randomized unknown-endpoint termination design. Methodologically, this framework links endpoint-specific indirect estimates with individual-level segment reconstruction while maintaining a clear distinction between cumulative endpoints and reconstructed segments; practically, it may provide complementary context for interpreting individual pacing execution and segmental mechanical performance.

## 2 Participants and methods

### 2.1 Participants

Twelve trained male rowers from the Fujian provincial rowing team participated in the study. Their mean sport-specific rowing training experience was 4.92 ± 1.38 years. The study was approved by the Science and Technology Ethics Committee of Jimei University (ethics review reference no. JMU202204030). All participants volunteered, were informed of the testing procedures, requirements, and potential risks, and provided written informed consent before participation.

The inclusion and pre-test requirements were as follows:

1. Participants had normal physiological function and had experienced no major injury during the month before testing.
2. No alcohol was consumed during the week before testing.
3. No strenuous exercise was performed on the day before testing.
4. Participants maintained their usual diet on the testing day and did not eat during the 2 h before testing.

All 12 participants completed every experimental trial. Participant characteristics are shown in **Table 1**.

**Table 1:** Participant characteristics.

Sample size	Age (years)	Height (cm)	Body mass (kg)	Training experience (years)
n=12	18.92 ± 1.51	191.25 ± 4.67	93.25 ± 12.11	4.92 ± 1.38

Values are mean ± standard deviation unless otherwise indicated; n=12.

### 2.2 Methods

#### 2.2.1 Experimental design and testing schedule

A within-participant repeated-measures design was used. The first trial was a 2000-m all-out rowing-ergometer reference test. Three pacing-matched endpoint trials were then completed in the fixed order of 500, 1000, and 1500 m. All testing was completed within 8 days, with 24–48 h between consecutive trials.

For each rower, cumulative completion time, mean power, and mean stroke rate at 500, 1000, and 1500 m were extracted from the 2000-m reference trial and used as individualized targets for the subsequent shorter endpoint trials. Participants were instructed to complete each endpoint trial according to their personal targets: the 500-m trial reproduced the first cumulative endpoint, the 1000-m trial reproduced the cumulative endpoint formed by the first two 500-m segments, and the 1500-m trial reproduced the cumulative endpoint formed by the first three 500-m segments.

The 2000-m reference trial recorded continuous mechanical and cardiorespiratory variables and established each rower’s individualized pacing targets. The shorter endpoint trials were used principally to obtain endpoint-specific post-exercise blood-lactate and recovery oxygen-uptake data at the corresponding endpoints under an approximately matched pacing context, thereby enabling cumulative glycolytic and phosphagen estimates. Because each shorter endpoint was completed only once, the analysis assessed pacing-replication accuracy rather than test–retest reliability.

Accordingly, the 500-, 1000-, and 1500-m trials were independent endpoint trials performed on different testing days. All trials were supervised by the same coach with rowing-training experience, who monitored technique and target execution and provided verbal encouragement.

#### 2.2.2 Experimental procedures and measurements

Before the first trial, participants completed a basic-information questionnaire and received information about the study purpose, procedures, and potential risks. On each testing day, participants completed a 10-min warm-up on a rowing ergometer (Model D, Concept2 Inc., USA), followed by 5 min of seated rest. The same ergometer was used for all trials. Participants were then fitted with a portable metabolic system (K4b², COSMED, Italy) and a heart-rate monitor (Polar Accurex Plus, Polar Electro Oy, Finland).

Respiratory gases were collected continuously throughout exercise and for 6 min after exercise using the portable metabolic system. Before each testing day, ambient pressure, gas concentrations, and volume were calibrated in accordance with the manufacturer’s instructions. Gas calibration used a certified mixture containing 15.00% O_2_ and 5.09% CO_2_, and volume calibration used a 3-L syringe.

Because peak post-exercise blood lactate may occur several minutes after intense exercise and its timing can vary with the exercise stimulus, serial recovery samples were collected to improve the likelihood of capturing the post-exercise peak response (Goodwin et al., 2007; Artioli et al., 2012). Seven 10-μL earlobe capillary blood samples were collected during each trial: before warm-up, immediately before the formal test, and at 1, 3, 5, 7, and 10 min after exercise. Blood lactate concentration was measured using a Biosen C-Line analyzer (EKF Diagnostics, Germany). Rating of perceived exertion was recorded immediately after each trial using the Borg 6–20 scale (Borg, 1982).

Split time, mean power, and mean stroke rate were extracted for the four actual consecutive 500-m segments of the 2000-m reference trial. Mean velocity was calculated as 500 m divided by split time.

#### 2.2.3 Calculation of estimated contributions from the three energy systems

Phosphagen, glycolytic, and aerobic contributions were estimated indirectly within the PCr–La–O_2_ framework from the fast component of excess post-exercise oxygen consumption, accumulated blood lactate, and cumulative oxygen uptake, respectively (de Campos Mello et al., 2009), using the following equations:


Estimated aerobic contribution (kJ) = net cumulative oxygen uptake during exercise (mL) × energy equivalent (J/mL)

Estimated glycolytic contribution (kJ) = net accumulated blood lactate (mmol/L) × oxygen–lactate conversion factor (mL/kg/mmol/L) × body mass (kg) × energy equivalent (J/mL)

Estimated phosphagen contribution (kJ) = fast component of excess post−exercise oxygen consumption (mL) × energy equivalent (J/mL)

The relative estimated contribution (%) of the phosphagen, glycolytic, and aerobic systems was calculated by dividing the absolute estimated contribution of each system by the summed estimated contribution of all three systems.

Net cumulative oxygen uptake during exercise was calculated by subtracting resting oxygen uptake, fixed at 4.5 mL·min^-1^·kg^-1^, from actual exercise oxygen uptake. Net accumulated blood lactate was calculated as peak post-exercise minus pre-exercise blood lactate. When the respiratory exchange ratio exceeded 1.0, an energy equivalent of 21.131 J·mL^-1^ O_2_ was used. The oxygen–lactate conversion factor was 3.0 mL O_2_·kg^-1^ for each 1 mmol·L^-1^ increase in blood lactate. EPOCfast was defined as actual cumulative oxygen uptake during the first 3 min of recovery minus cumulative slow-component oxygen uptake over the same interval; the slow component for the first 3 min was estimated by back-extrapolating the oxygen-uptake curve from recovery minutes 3–6 (Li and Mao, 2014; Li et al., 2017). Respiratory gases were recorded for 6 min after each trial and EPOCfast was calculated using this procedure.

#### 2.2.4 Statistical analysis of pacing-replication accuracy

At the 500-, 1000-, and 1500-m endpoints, each rower’s target completion time, mean power, and mean stroke rate were paired with the corresponding achieved values. Bias was defined as achieved minus target and is reported as mean bias with a two-sided 95% confidence interval. Mean absolute error (MAE), mean absolute percentage error (MAPE), root-mean-square error, and maximum absolute error were also calculated. The Shapiro–Wilk test was used to assess the distribution of paired differences. Mean systematic bias was evaluated using a one-sample t test of the paired differences, with the Wilcoxon signed-rank test used as a nonparametric sensitivity analysis. Holm correction was applied separately to the nine endpoint-by-metric tests for the t tests and Wilcoxon tests. Agreement was described using Lin’s concordance correlation coefficient and Bland–Altman mean bias with 95% limits of agreement. Because no equivalence bounds with physiological or practical meaning had been prespecified, equivalence testing was not performed and p>0.05 was not interpreted as statistical equivalence. The primary analysis included all 36 paired records. To assess the influence of two larger 1500-m deviations, a sensitivity analysis excluding S03 and S04 was also conducted; this was supplementary and did not replace the primary analysis retaining all observations.

#### 2.2.5 Statistical analysis of consecutive 500-m kinematics

Split time, mean power, mean stroke rate, and mean velocity across the four actual consecutive 500-m segments of the original 2000-m reference trial were each analyzed using one-way repeated-measures analysis of variance. Sphericity was evaluated using Mauchly’s test, with Greenhouse–Geisser correction when required. Holm-adjusted pairwise comparisons followed significant omnibus effects, and partial η² was reported as the omnibus effect size. Friedman and Wilcoxon analyses were used as nonparametric sensitivity checks.

#### 2.2.6 Physiological and performance variables

Physiological and performance variables from the four endpoint trials are reported to characterize the testing conditions. Unless an inferential comparison is explicitly stated in the Results, these variables are interpreted descriptively. Previously established pairwise comparisons for EPOCfast and RPE were retained from the original analysis.

#### 2.2.7 Statistical analysis of energy-system contributions

All energy-system analyses were conducted using participant-level data from the 12 rowers. Cumulative endpoint data and reconstructed 500-m segment data were treated as two separate analytical levels; group means were never subtracted to replace within-rower reconstruction. The primary outcomes were absolute estimated phosphagen, glycolytic, and aerobic contributions (kJ), with their sum treated as a supplementary outcome. Relative estimated contributions were secondary outcomes; reconstructed segment proportions were recalculated from the three absolute contributions within each segment. All negative reconstructed values were retained unchanged and were not deleted, set to zero, converted to absolute values, imputed, or transformed. Data are presented as mean ± standard deviation, with two-sided 95% confidence intervals for each condition mean. For each outcome, a one-way repeated-measures analysis of variance with four levels compared the four cumulative endpoints (500, 1000, 1500, and 2000 m) or the four reconstructed segments (0–500, 500–1000, 1000–1500, and 1500–2000 m). Shapiro–Wilk tests were used to assess normality of condition values, model residuals, and paired differences. Sphericity was evaluated using Mauchly’s test, with Greenhouse–Geisser correction of the degrees of freedom and p value when sphericity was rejected. When the omnibus effect was significant, all six paired comparisons were conducted and Holm correction was applied within each outcome. Omnibus effects are reported as partial η². Given the sample size of 12 and the presence of negative reconstructed values, Friedman tests and Kendall’s W were also used as sensitivity analyses for the omnibus effects. All tests were two-sided with α=0.05; no single multiplicity correction was applied across different energy systems or across different outcome models. Exact p values are reported except when p<0.001. Holm-adjusted pairwise-comparison p values for the primary absolute-contribution outcomes are provided in **Appendix Table A**.

## 3 Results

### 3.1 Pacing-replication accuracy

At 500 m, MAE values for time, power, and stroke rate were 0.050 s, 0.667 W, and 0.750 spm, with MAPE values of 0.053%, 0.157%, and 2.483%, and Lin’s concordance correlation coefficients of 0.999, 0.999, and 0.761, respectively. At 1000 m, the corresponding MAE values were 0.092 s, 0.917 W, and 0.396 spm, MAPE values were 0.048%, 0.232%, and 1.370%, and Lin’s concordance correlation coefficients were 1.000, 0.999, and 0.955.

At 1500 m, MAE values for time, power, and stroke rate were 1.258 s, 5.083 W, and 0.359 spm, with MAPE values of 0.431%, 1.339%, and 1.239%. The corresponding Lin concordance correlation coefficients were 0.933, 0.928, and 0.954. Bland–Altman 95% limits of agreement for time and power were −5.723 to 5.406 s and −22.323 to 22.156 W, respectively, and were wider than those at 500 and 1000 m. No stable mean or median systematic bias was detected across the nine outcomes after Holm adjustment. At 1500 m, S03 and S04 had time deviations of +6.6 and −6.7 s and power deviations of −27 and +26 W, respectively. In the sensitivity analysis excluding these two records, time MAE decreased to 0.180 s and power MAE to 0.800 W, but this analysis did not replace the primary analysis retaining all observations. Because no equivalence bounds had been prespecified, statistical equivalence was not concluded. Complete Bland–Altman plots for the nine endpoint-by-metric combinations are provided in **Supplementary Figures S1**–**S9**. Complete pacing-replication accuracy results are presented in **Table 2**.

**Table 2:** Pacing-replication accuracy between individualized 2000-m targets and achieved values in the shorter endpoint trials.

Endpoint	Variable	Mean bias [95% CI]	MAE	MAPE (%)	RMSE	Lin CCC	Bland–Altman 95% LoA
500 m	Time (s)	0.017 [-0.043, 0.076]	0.050	0.053	0.091	0.999	[-0.167, 0.200]
500 m	Power (W)	-0.167 [-0.925, 0.592]	0.667	0.157	1.155	0.999	[-2.506, 2.172]
500 m	Stroke rate (spm)	-0.083 [-0.716, 0.550]	0.750	2.483	0.957	0.761	[-2.036, 1.869]
1000 m	Time (s)	0.008 [-0.066, 0.082]	0.092	0.048	0.112	1.000	[-0.220, 0.237]
1000 m	Power (W)	-0.500 [-1.204, 0.204]	0.917	0.232	1.173	0.999	[-2.671, 1.671]
1000 m	Stroke rate (spm)	0.146 [-0.129, 0.421]	0.396	1.370	0.439	0.955	[-0.702, 0.993]
1500 m	Time (s)	-0.158 [-1.962, 1.646]	1.258	0.431	2.723	0.933	[-5.723, 5.406]
1500 m	Power (W)	-0.083 [-7.293, 7.126]	5.083	1.339	10.864	0.928	[-22.323, 22.156]
1500 m	Stroke rate (spm)	0.155 [-0.115, 0.425]	0.359	1.239	0.435	0.954	[-0.677, 0.987]

Bias = achieved − target; CI, two-sided 95% confidence interval for mean bias; MAE, mean absolute error; MAPE, mean absolute percentage error; RMSE, root-mean-square error; CCC, Lin’s concordance correlation coefficient; LoA, Bland–Altman 95% limits of agreement; n=12 at each endpoint. The primary analysis retained all 36 original paired records. Shapiro–Wilk tests indicated non-normality for 500-m time, 500-m power, 1000-m time, 1500-m time, and 1500-m power differences; all nine Wilcoxon signed-rank tests were non-significant after Holm correction, consistent with the t-test conclusions. No equivalence bounds were prespecified; statistical equivalence was therefore not concluded.

### 3.2 Consecutive 500-m kinematics in the original 2000-m reference trial

Repeated-measures analyses showed significant segment effects for split time, mean power, mean stroke rate, and mean velocity. The 0–500-m segment had the shortest split time and the highest mean power and mean velocity, whereas the 1500–2000-m segment had the highest mean stroke rate. Holm-adjusted *post hoc* comparisons supported these opening- and finishing-phase differences, and nonparametric sensitivity analyses were directionally consistent. These values were obtained from the four actual consecutive 500-m segments of the 2000-m reference trial and must be distinguished from both cumulative endpoint energy estimates and difference-based reconstructed 500-m energy contributions. Complete kinematic results across the four actual consecutive 500-m segments are presented in **Table 3**.

**Table 3:** Kinematics across the four actual consecutive 500-m segments of the original 2000-m reference trial.

Segment	Split time (s)	Mean power (W)	Mean stroke rate (spm)	Mean velocity (m/s)
0–500 m	95.17 ± 2.81	407.8 ± 34.8	29.6 ± 1.7	5.26 ± 0.15
500–1000 m	97.83 ± 2.62	375.2 ± 29.6	28.8 ± 1.7	5.11 ± 0.14
1000–1500 m	98.88 ± 2.79	363.8 ± 31.1	29.2 ± 1.7	5.06 ± 0.14
1500–2000 m	97.62 ± 2.00	377.1 ± 22.8	31.9 ± 2.4	5.12 ± 0.10
Omnibus test	F(2.05,22.55)=14.767, p<0.001, ηp²=0.573	F(2.04,22.47)=15.770, p<0.001, ηp²=0.589	F(1.77,19.44)=20.332, p<0.001, ηp²=0.649	F(2.05,22.57)=15.277, p<0.001, ηp²=0.581

Values are mean ± standard deviation; n=12. Omnibus tests are one-way repeated-measures ANOVA results with Greenhouse–Geisser correction where required. Holm-adjusted *post hoc* comparisons are described in the text. ηp², partial eta squared.

### 3.3 Physiological and performance variables at the four endpoint trials

Group-level physiological and performance variables for the four endpoint trials are shown in **Table 4**. Mean velocity, mean stroke rate, mean power, peak post-exercise heart rate, peak post-exercise blood lactate, net blood-lactate accumulation, peak oxygen uptake, and cumulative oxygen uptake are presented descriptively unless an inferential comparison is explicitly stated. At the 2000-m endpoint, peak post-exercise heart rate and peak oxygen uptake were 185.67 ± 7.14 bpm and 69.57 ± 5.80 mL·min^-1^·kg^-1^, respectively.

**Table 4:** Physiological and performance variables in the four endpoint trials.

Variable	Unit	500-m endpoint	1000-m endpoint	1500-m endpoint	2000-m endpoint
Mean velocity	m/s	5.26 ± 0.15	5.18 ± 0.14	5.15 ± 0.13	5.17 ± 0.12
Mean stroke rate	spm	33.33 ± 1.97	29.00 ± 1.60	29.25 ± 1.60	29.33 ± 1.23
Mean power	W	407.92 ± 34.92	391.00 ± 31.35	382.17 ± 30.00	388.42 ± 25.90
Peak post-exercise heart rate	bpm	149.67 ± 16.39	168.50 ± 8.43	173.67 ± 9.83	185.67 ± 7.14
Peak post-exercise blood lactate	mmol/L	5.73 ± 0.64	9.63 ± 0.88	10.59 ± 0.10	12.68 ± 1.12
Net blood-lactate accumulation	mmol/L	4.73 ± 0.60	8.63 ± 0.91	9.59 ± 0.90	11.68 ± 1.02
Peak oxygen uptake	mL·min^-1^·kg^-1^	56.12 ± 6.05	61.70 ± 6.34	63.44 ± 5.76	69.57 ± 5.80
Cumulative oxygen uptake	L	3.87 ± 0.57	9.45 ± 1.32	14.29 ± 1.90	20.38 ± 2.38
EPOCfast	L	0.862 ± 0.276	1.019 ± 0.393	1.340 ± 0.386	1.473 ± 0.323
RPE	6–20	12.42 ± 1.08	14.50 ± 0.91	16.33 ± 1.07	16.92 ± 2.19

Values are mean ± standard deviation; n=12. EPOCfast, fast component of excess post-exercise oxygen consumption; RPE, Borg 6–20 rating of perceived exertion. Statistical comparisons explicitly reported in Section 3.3 should be distinguished from descriptive trends in the remaining variables.

For EPOCfast, the 1500-m value was significantly higher than the 500- and 1000-m values, whereas the 500–1000-m and 1500–2000-m comparisons were not significant. For RPE, the 1000-m value was significantly higher than the 500-m value, and the 1500-m value was significantly higher than both the 500- and 1000-m values; the 1500–2000-m comparison was not significant. Other apparent endpoint trends in **Table 4** should therefore be interpreted descriptively rather than as inferentially established differences.

### 3.4 Estimated energy-system contributions at four cumulative endpoints

Significant endpoint effects were found for absolute phosphagen, glycolytic, aerobic, and total estimated contributions and for the relative estimated contributions of the three systems (**Table 5**). For absolute contributions, glycolytic, aerobic, and total estimated contributions increased with endpoint distance. Cumulative phosphagen contribution differed only between selected more distant endpoints, and none of the three adjacent-endpoint comparisons was significant. For relative contributions, phosphagen and glycolytic percentages generally decreased, whereas aerobic percentage generally increased. Complete descriptive values, 95% confidence intervals, omnibus tests, and Holm-adjusted comparison groupings are shown in **Table 5**. All results represent cumulative estimates at the end of independent endpoint trials and do not represent independent 500-m segments within a continuous 2000-m effort. Individual cumulative trajectories for the three systems are shown in **Supplementary Figures S10**–**S12**.

**Table 5:** Absolute and relative estimated energy-system contributions at four cumulative endpoints.

System/outcome	500 m	1000 m	1500 m	2000 m	Omnibus test
Phosphagen (kJ)	18.21 ± 5.83 [14.51, 21.91]a	21.54 ± 8.30 [16.26, 26.81]ab	28.32 ± 8.16 [23.13, 33.50]bc	31.12 ± 6.82 [26.79, 35.46]c	F(3,33)=9.420, p<0.001, ηp²=0.461
Phosphagen (%)	14.15 ± 3.91% [11.67, 16.63]a	7.87 ± 2.71% [6.15, 9.59]bc	7.29 ± 1.76% [6.17, 8.41]b	5.88 ± 1.10% [5.18, 6.57]c	F(1.923,21.150)=23.731, p<0.001, ηp²=0.683
Glycolytic (kJ)	27.55 ± 6.74 [23.27, 31.84]a	49.72 ± 9.18 [43.89, 55.56]b	55.09 ± 8.69 [49.56, 60.61]c	66.71 ± 7.14 [62.17, 71.25]d	F(3,33)=189.054, p<0.001, ηp²=0.945
Glycolytic (%)	21.71 ± 5.41% [18.27, 25.15]a	18.59 ± 4.21% [15.92, 21.27]b	14.57 ± 3.44% [12.39, 16.75]c	12.80 ± 2.32% [11.33, 14.28]d	F(1.765,19.416)=56.614, p<0.001, ηp²=0.837
Aerobic (kJ)	81.87 ± 12.09 [74.19, 89.56]a	199.74 ± 27.84 [182.05, 217.43]b	301.92 ± 40.14 [276.42, 327.43]c	430.55 ± 50.40 [398.53, 462.57]d	F(1.734,19.070)=561.620, p<0.001, ηp²=0.981
Aerobic (%)	64.14 ± 6.12% [60.25, 68.03]a	73.54 ± 4.31% [70.80, 76.28]b	78.14 ± 3.58% [75.86, 80.42]c	81.32 ± 2.48% [79.75, 82.90]d	F(1.909,21.000)=81.357, p<0.001, ηp²=0.881
Total estimated contribution (kJ)	127.64 ± 14.69 [118.31, 136.97]a	271.01 ± 29.41 [252.32, 289.69]b	385.33 ± 38.64 [360.77, 409.88]c	528.39 ± 48.09 [497.83, 558.94]d	F(3,33)=625.562, p<0.001, ηp²=0.983

Values are mean ± SD, with two-sided 95% confidence intervals for the means in brackets; n=12. Values within a row that do not share a lowercase letter differ significantly after Holm adjustment (two-sided p<0.05). ηp², partial eta squared. Units are specified in row labels; relative contributions are percentages at the corresponding cumulative endpoint. All results are cumulative endpoint estimates and do not represent independent 500-m segments.

### 3.5 Reconstructed absolute estimated energy-system contributions across successive 500-m segments

Within-rower reconstruction showed significant segment effects for absolute phosphagen, glycolytic, and aerobic contributions (**Table 6**). Estimated phosphagen contribution during 0–500 m was higher than during each of the other three segments, whereas the latter three did not differ from one another. Estimated glycolytic contribution was higher during the first two segments than during the final two segments, with no difference within the first pair or within the final pair. Estimated aerobic contribution was lower during 0–500 m than during each of the other three segments, which did not differ from one another. Although the parametric omnibus test for total estimated contribution was significant, no Holm-adjusted pairwise comparison was significant and the Friedman sensitivity test was non-significant; therefore, no robust segmental difference in total estimated contribution was concluded. Thirteen negative cells were retained: 11 for phosphagen, 2 for glycolytic, and none for aerobic contribution. All negative values were retained unchanged. Individual reconstructed trajectories for the three systems are shown in **Figures 1**–**3**.

**Table 6:** Reconstructed absolute estimated energy-system contributions across successive 500-m segments (kJ).

System/outcome	0–500 m	500–1000 m	1000–1500 m	1500–2000 m	Omnibus test
Phosphagen	18.21 ± 5.83 [14.51, 21.91]a	3.33 ± 9.33 [-2.60, 9.26]b Negative values=5	6.78 ± 11.71 [-0.66, 14.22]b Negative values=2	2.81 ± 7.52 [-1.97, 7.58]b Negative values=4	F(3,33)=6.115, p=0.002, ηp²=0.357
Glycolytic	27.55 ± 6.74 [23.27, 31.84]a	22.17 ± 5.15 [18.90, 25.44]a	5.36 ± 6.37 [1.31, 9.41]b Negative values=2	11.63 ± 5.07 [8.41, 14.85]b	F(3,33)=28.904, p<0.001, ηp²=0.724
Aerobic	81.87 ± 12.09 [74.19, 89.56]a	117.87 ± 21.30 [104.34, 131.40]b	102.18 ± 21.57 [88.48, 115.88]b	128.62 ± 26.80 [111.60, 145.65]b	F(3,33)=12.949, p<0.001, ηp²=0.541
Total estimated contribution	127.64 ± 14.69 [118.31, 136.97]a	143.37 ± 23.00 [128.75, 157.98]a	114.32 ± 26.09 [97.74, 130.90]a	143.06 ± 28.93 [124.68, 161.44]a	F(3,33)=4.154, p=0.013, ηp²=0.274; Friedman p=0.117

Values are mean ± SD, with two-sided 95% confidence intervals for the means in brackets; n=12. Values within a row that do not share a lowercase letter differ significantly after Holm adjustment (two-sided p<0.05). ηp², partial eta squared. “Negative values” indicates the number of participant-level reconstructed values below zero in that condition; all negative values were retained unchanged.

**Figure 1:**
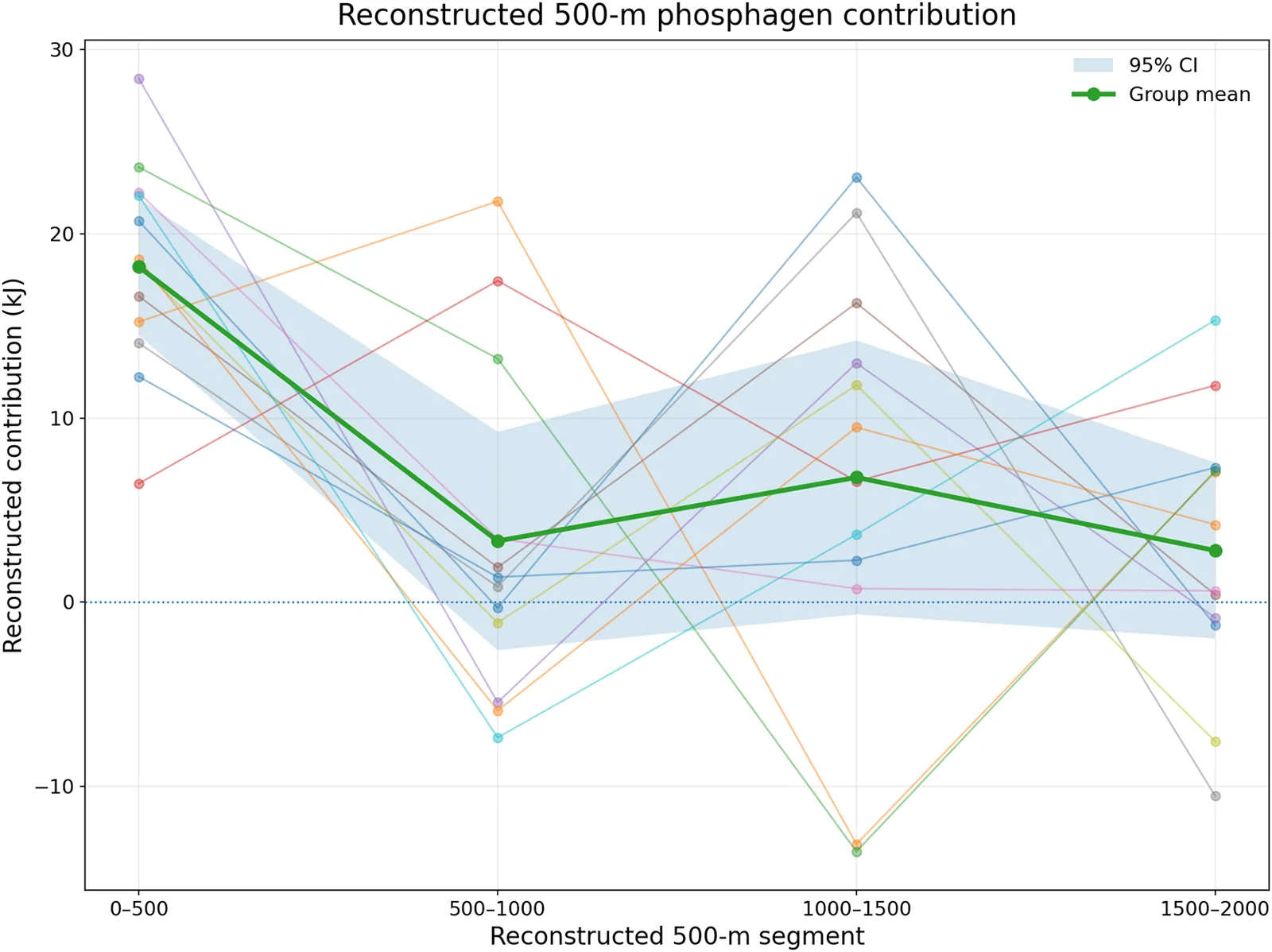
Individually reconstructed estimated phosphagen contribution across four successive 500-m segments in 12 rowers. Thin lines represent individual rowers, the thick line represents the group mean, and the shaded area represents the 95% confidence interval of the group mean.

**Figure 2:**
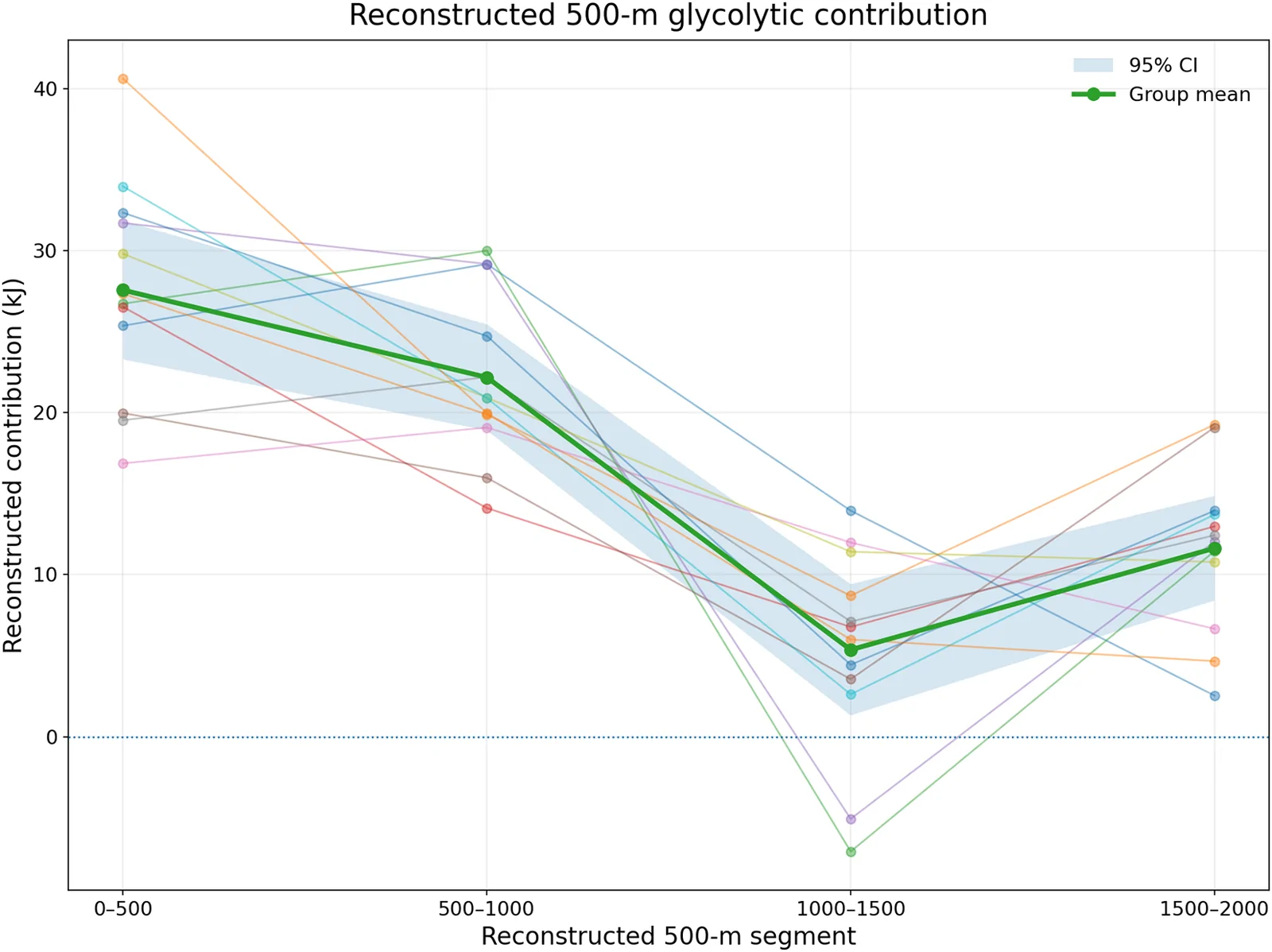
Individually reconstructed estimated glycolytic contribution across four successive 500-m segments in 12 rowers. Thin lines represent individual rowers, the thick line represents the group mean, and the shaded area represents the 95% confidence interval of the group mean.

**Figure 3:**
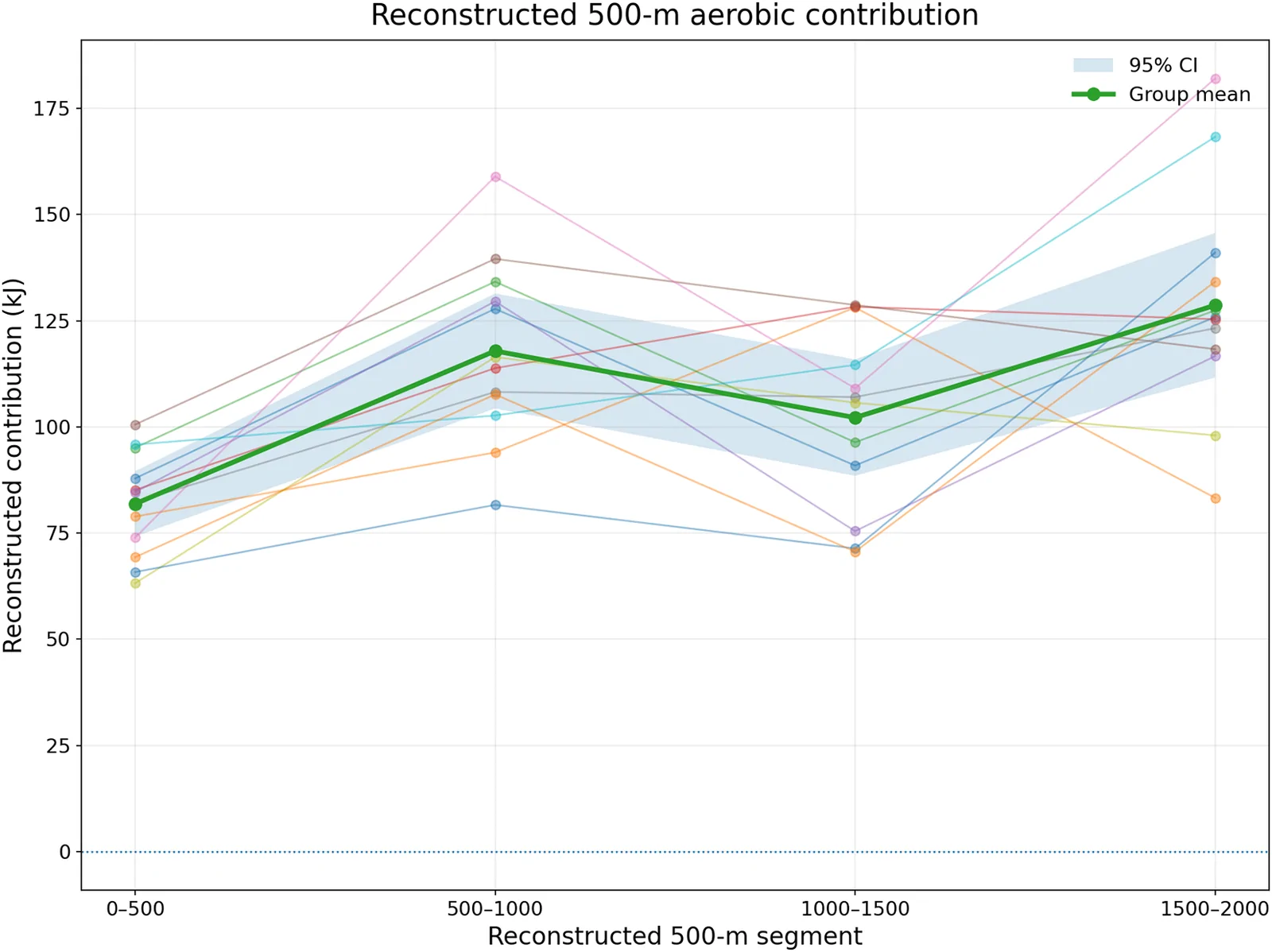
Individually reconstructed estimated aerobic contribution across four successive 500-m segments in 12 rowers. Thin lines represent individual rowers, the thick line represents the group mean, and the shaded area represents the 95% confidence interval of the group mean.

### 3.6 Reconstructed relative estimated energy-system contributions across successive 500-m segments

All three relative estimated contributions showed significant omnibus segment effects (**Table 7**). The overall pattern was a higher phosphagen percentage during 0–500 m than during selected later segments, higher glycolytic percentages during the first two segments than during the final two segments, and a lower aerobic percentage during 0–500 m than during the other three segments. Because reconstructed absolute contributions could be negative, the corresponding percentages could also be negative or exceed 100%. Relative contributions were therefore interpreted as secondary outcomes, with the absolute-contribution analysis given priority.

**Table 7:** Reconstructed relative estimated energy-system contributions across successive 500-m segments (%).

System/outcome	0–500 m	500–1000 m	1000–1500 m	1500–2000 m	Omnibus test
Phosphagen	14.15 ± 3.91% [11.67, 16.63]a	2.07 ± 6.67% [-2.17, 6.31]b Negative values=5	5.26 ± 10.77% [-1.59, 12.10]ab Negative values=2	1.46 ± 5.38% [-1.96, 4.88]b Negative values=4	F(1.674,18.410)=6.316, p=0.011, ηp²=0.365
Glycolytic	21.71 ± 5.41% [18.27, 25.15]a	15.76 ± 3.99% [13.22, 18.30]b	4.03 ± 6.14% [0.13, 7.93]c Negative values=2	8.52 ± 4.43% [5.70, 11.33]c	F(3,33)=28.661, p<0.001, ηp²=0.723
Aerobic	64.14 ± 6.12% [60.25, 68.03]a	82.17 ± 6.25% [78.20, 86.14]b	90.71 ± 14.16% [81.71, 99.70]bc	90.02 ± 7.06% [85.54, 94.51]c	F(1.560,17.160)=18.976, p<0.001, ηp²=0.633

Values are mean ± SD, with two-sided 95% confidence intervals for the means in brackets; n=12. Values within a row that do not share a lowercase letter differ significantly after Holm adjustment (two-sided p<0.05). ηp², partial eta squared. “Negative values” indicates the number of participant-level reconstructed values below zero in that condition; all negative values were retained unchanged. Because participant-level reconstructed absolute contributions could be negative, percentages could be negative or exceed 100% and are interpreted only as secondary outcomes.

## 4 Discussion

### 4.1 Main findings and contribution

The principal contribution of the present study lies in integrating an individualized 2000-m pacing template, verification of pacing-replication accuracy during shorter endpoint trials, cumulative endpoint estimates of energy-system contributions, and within-rower reconstruction of successive 500-m segments within a single analytical framework. By explicitly distinguishing cumulative endpoint estimates from reconstructed 500-m segments, this framework allows estimated energetic patterns across race phases to be interpreted alongside actual segmental kinematics and compared descriptively with Yan’s randomized unknown-endpoint termination design.

These reconstructed patterns are physiologically consistent with the differing temporal characteristics of the three energy systems during high-intensity exercise. The phosphagen system can provide ATP at a high rate at exercise onset but has limited capacity, making substantial early involvement compatible with the high power demand of the rowing start (Bogdanis et al., 1996). As intense exercise continues beyond the initial seconds, glycolytic metabolism provides an important non-oxidative contribution, while the progressive increase in oxygen uptake shifts a greater proportion of energy provision toward oxidative metabolism as exercise duration increases (Medbø and Tabata, 1989). In 2000-m rowing specifically, previous studies have consistently demonstrated substantial aerobic predominance across the complete performance while retaining meaningful anaerobic contributions to the high power demands of the event (Pripstein et al., 1999; de Campos Mello et al., 2009). López-Sánchez et al. (2018) further reported distance-dependent physiological and metabolic responses during shorter rowing trials matched to characteristics of an initial 2000-m performance. The present reconstructed pattern should therefore be interpreted within this established physiological framework, while its added value lies in resolving estimated energy-system contributions across successive 500-m segments under individualized pacing-matched conditions.

### 4.2 Interpretive boundary between cumulative endpoints and reconstructed 500-m segments

Interpretation should distinguish cumulative endpoint estimates from the reconstructed 500-m values. The latter are mathematical reconstructions derived from differences between cumulative endpoint estimates rather than direct physiological measurements of independent race segments. Because the endpoint trials were performed independently on different testing days, the reconstructed values should be interpreted as estimates under a known-endpoint design rather than as direct measurements of physiological events occurring within one uninterrupted 2000-m performance.

Occasional negative reconstructed values should be interpreted as a consequence of differencing two indirectly estimated cumulative endpoints rather than as physiologically negative energy release. Such values therefore illustrate the uncertainty that can be amplified when independent cumulative estimates are differenced at the individual level.

### 4.3 Reconstructed segmental pattern of phosphagen contribution

The early prominence of the estimated phosphagen contribution is physiologically compatible with the high immediate power demand of the rowing start and the rapid ATP requirement before oxidative metabolism reaches a greater contribution. Phosphocreatine availability is particularly important during the first seconds of high-power exercise (Bogdanis et al., 1996), while rowing pacing studies have consistently identified a fast opening phase as a characteristic feature of 2000-m performance (Garland, 2005). These observations support interpreting the opening reconstruction as a period of substantial rapid-energy demand without treating the reconstructed value as a direct intramuscular measurement.

### 4.4 Reconstructed segmental pattern of glycolytic contribution

The reconstructed glycolytic pattern is consistent with previous evidence that early high-power exercise places substantial demand on non-oxidative glycolysis, whereas the relative contribution of oxidative metabolism increases as severe-intensity exercise continues (Medbø and Tabata, 1989). In rowing, this transition occurs within the broader context of an event that remains predominantly aerobic overall while still requiring meaningful anaerobic support for high power production (Pripstein et al., 1999; de Campos Mello et al., 2009). Because the glycolytic estimate is based on blood-lactate accumulation measured after independently terminated trials, it is most appropriately interpreted as an indirect reconstruction of the segmental pattern.

### 4.5 Reconstructed segmental pattern of aerobic contribution

The lower opening-phase aerobic estimate is consistent with the time required for oxygen uptake to rise after exercise onset, whereas sustained oxidative demand thereafter accords with the established aerobic predominance of 2000-m rowing (Ingham et al., 2007; Boillet et al., 2022; Pripstein et al., 1999). This interpretation is also compatible with broader severe-intensity exercise physiology, in which oxygen uptake progressively approaches the level required by the task while finite non-oxidative resources contribute to the work performed above sustainable metabolic rates.

### 4.6 Integration of the three systems and total estimated contribution

The actual consecutive 500-m kinematics provide mechanical context for interpreting the reconstructed energetic pattern. The high mechanical demand of the opening phase is compatible with substantial immediate energy requirements, whereas changes in stroke rate later in the trial should be interpreted together with the accompanying power and velocity responses rather than as an isolated indicator of performance. More broadly, the observed pattern can be considered within the severe-intensity/critical-power framework, which emphasizes interactions among aerobic power, finite non-oxidative work capacity, and pacing regulation (Borges et al., 2025; Possamai et al., 2024; Astridge et al., 2024). The absence of robust pairwise or nonparametric evidence for differences in total reconstructed contribution further suggests that changes in the composition of the three estimated systems were more evident than changes in total estimated contribution.

### 4.7 Comparison with Yan’s randomized unknown-endpoint termination design

The broad qualitative correspondence between the present pacing-matched reconstruction and Yan’s randomized unknown-endpoint approach provides useful cross-design context for interpreting the segmental patterns. Both approaches identified prominent early phosphagen involvement and greater glycolytic contribution in the first half of the task. However, aerobic and total-contribution findings were not fully consistent. This comparison should not be regarded as evidence of methodological equivalence or validation because sample composition, endpoint expectancy, trial structure, pacing control, and the procedures used to generate segment-level estimates differed between the two designs.

### 4.8 Relationship to previous endpoint-trial studies

López-Sánchez et al. (2018) provide a relevant methodological precedent for pacing-matched endpoint trials because their participants completed shorter trials while reproducing performance characteristics from an initial 2000-m effort. The present design extended this concept by matching individualized cumulative time, mean power, and mean stroke rate and by formally quantifying target–achieved error. The small absolute errors at 500 and 1000 m and the larger individual deviations observed at 1500 m reinforce the importance of evaluating pacing replication with absolute-error and agreement metrics rather than relying only on group mean bias or correlation.

Li et al. (2017) provide complementary evidence from independent self-paced all-out rowing trials of different durations, but those trials were not designed to reproduce the cumulative pacing profile of a prior 2000-m performance. Their full-duration phosphagen estimate was broadly similar to the present 2000-m cumulative estimate, whereas direct numerical comparison at shorter durations is limited by differences in sample composition, endpoint definition, trial order, and pacing instructions. These design differences underline why cumulative endpoint estimates and reconstructed race segments should not be treated interchangeably across studies.

### 4.9 Limitations and future directions

Several design limitations should be considered. The 500-, 1000-, and 1500-m trials were known-endpoint tests performed on separate days and in a fixed order. Although individualized pacing matching reduced performance differences among trials, it could not eliminate endpoint expectancy, day-to-day variability, or potential order and learning effects. Each shorter endpoint trial was completed only once; therefore, the target–actual analyses quantify pacing-replication accuracy rather than test–retest reliability. In addition, the relatively small and homogeneous sample comprised only 12 trained male rowers from a single provincial team. This limits the precision and generalizability of the findings, which should therefore be interpreted primarily within populations with characteristics similar to those of the present participants.

The energy-system results should also be interpreted within the limits of the estimation approach. The PCr–La–O_2_ framework indirectly estimates energy-system contributions from oxygen uptake, post-exercise blood lactate, and recovery oxygen uptake and does not constitute a direct measurement of intramuscular energy metabolism. Successive 500-m segments were further reconstructed by differencing adjacent cumulative endpoint estimates; consequently, between-trial measurement variability may be amplified during subtraction and may generate occasional negative reconstructed values. Such values reflect uncertainty in the indirect estimation and differencing procedure rather than physiologically negative energy release.

Future studies should examine the reproducibility of individualized pacing-matched endpoint trials and the resulting 500-m reconstructions and determine whether comparable segmental patterns are obtained when fixed known-endpoint trials are contrasted with randomized or counterbalanced testing orders and unknown-endpoint termination designs. Larger cohorts including female rowers and athletes from different competitive settings are needed to evaluate generalizability. Future studies should also prospectively characterize training and performance status using standardized information such as recent competition level, primary boat class, rowing-specific training history, weekly rowing and resistance-training exposure, and objective rowing-performance measures. Longitudinal work could further determine whether reconstructed 500-m energetic profiles are sensitive to training adaptation and whether changes in these profiles correspond to changes in 2000-m performance and actual segmental kinematics.

### 4.10 Practical implications

For trained male provincial-team rowers, the combined framework may be useful for interpreting whether prescribed 500-, 1000-, and 1500-m checkpoints are reproduced as intended and for examining segmental mechanical performance alongside reconstructed energetic patterns. These reconstructed profiles are best viewed as individualized monitoring hypotheses rather than direct prescriptions for manipulating training intensity or volume.

In the broader context of paddle sports, recent work in international-level canoeists and kayakers has also highlighted load–power relationships in upper-body pushing and pulling exercises as potentially useful complementary information for individualized strength and power assessment (Crisafulli et al., 2025a). Because that evidence was obtained in canoeists and kayakers rather than rowers, it should not be directly extrapolated as a rowing-specific training prescription.

## 5 Conclusions

Individualized known-endpoint trials reproduced each rower’s 2000-m pacing template with good accuracy. Replication was most stable at 500 and 1000 m, whereas the 1500-m endpoint was more susceptible to individual deviations. This design provided post-exercise blood-lactate and recovery oxygen-uptake data at each cumulative endpoint, enabling indirect energy-system estimation and within-rower reconstruction of successive 500-m contributions. Reconstruction showed that estimated phosphagen contribution was concentrated primarily during 0–500 m, estimated glycolytic contribution primarily within the first 1000 m, and estimated aerobic contribution remained high after the opening segment; no robust segmental difference in total estimated contribution was supported.

Together with the actual consecutive 500-m kinematics from the 2000-m reference trial, the study links pacing-replication accuracy, cumulative endpoint estimation, and successive-segment reconstruction and may provide complementary information for interpreting 2000-m pacing execution and segment-specific energetic demands. These reconstructed segment values remain indirect estimates and should not be interpreted as direct physiological measurements of continuous race phases.

## Data Availability

The original contributions presented in the study are included in the article/**Supplementary Material**. Further inquiries can be directed to the corresponding author.

## Appendix Table A

**Table A:** Holm-adjusted pairwise-comparison p values for the primary absolute estimated contribution outcomes.

Level/outcome	Comparison 1	Comparison 2	Comparison 3	Comparison 4	Comparison 5	Comparison 6	Order
Cumulative—phosphagen	0.444	0.018	0.003	0.210	0.020	0.444	C1–C2, C1–C3, C1–C4, C2–C3, C2–C4, C3–C4
Cumulative—glycolytic	<0.001	<0.001	<0.001	0.014	<0.001	<0.001	C1–C2, C1–C3, C1–C4, C2–C3, C2–C4, C3–C4
Cumulative—aerobic	<0.001	<0.001	<0.001	<0.001	<0.001	<0.001	C1–C2, C1–C3, C1–C4, C2–C3, C2–C4, C3–C4
Cumulative—total estimated contribution	<0.001	<0.001	<0.001	<0.001	<0.001	<0.001	C1–C2, C1–C3, C1–C4, C2–C3, C2–C4, C3–C4
Reconstructed—phosphagen	0.012	0.049	0.002	1.000	1.000	1.000	C1–C2, C1–C3, C1–C4, C2–C3, C2–C4, C3–C4
Reconstructed—glycolytic	0.068	<0.001	<0.001	<0.001	0.003	0.068	C1–C2, C1–C3, C1–C4, C2–C3, C2–C4, C3–C4
Reconstructed—aerobic	<0.001	0.018	<0.001	0.152	0.223	0.092	C1–C2, C1–C3, C1–C4, C2–C3, C2–C4, C3–C4
Reconstructed—total estimated contribution	0.207	0.373	0.294	0.066	0.977	0.177	C1–C2, C1–C3, C1–C4, C2–C3, C2–C4, C3–C4

Values are Holm-adjusted p values for the primary absolute-contribution outcomes. For cumulative endpoints, C1–C4 denote 500, 1000, 1500, and 2000 m; for reconstructed segments, C1–C4 denote 0–500, 500–1000, 1000–1500, and 1500–2000 m.
