# Supplementary material for: Cumulative endpoint and reconstructed 500-m energy-system contributions from individualized pacing-matched trials in 2000-m rowing ergometry
Source: Front Physiol. 2026 Sep 29;17:1840466. doi: 10.3389/fphys.2026.1840466 (PMC13657997; doi:10.3389/fphys.2026.1840466)
Supplement: Supplementary file 1 [file SupplementaryFile1.docx]

**Supplementary Material**

**Cumulative Endpoint and Reconstructed 500-m Energy-System Contributions From Individualized Pacing-Matched Trials in 2000-m Rowing Ergometry**

*Supplementary Figures S1–S12*

Supplementary Figures S1–S9 present Bland–Altman analyses of pacing-replication accuracy for cumulative time, mean power, and mean stroke rate at the 500-, 1000-, and 1500-m endpoints. Supplementary Figures S10–S12 present participant-level cumulative endpoint trajectories for the indirectly estimated phosphagen, glycolytic, and aerobic contributions. All analyses include 12 participants. The former Supplementary Table S1 is not included because the actual consecutive 500-m kinematic results have been incorporated into the main manuscript as Table 3.

Supplementary Figure S1. Bland–Altman plot for cumulative time replication at the 500-m endpoint. The y-axis represents achieved minus target time. Solid and dashed horizontal lines denote mean bias and the 95% limits of agreement, respectively.

Supplementary Figure S2. Bland–Altman plot for mean power replication at the 500-m endpoint. The y-axis represents achieved minus target power. Solid and dashed horizontal lines denote mean bias and the 95% limits of agreement, respectively.

Supplementary Figure S3. Bland–Altman plot for mean stroke-rate replication at the 500-m endpoint. The y-axis represents achieved minus target stroke rate. Solid and dashed horizontal lines denote mean bias and the 95% limits of agreement, respectively.

Supplementary Figure S4. Bland–Altman plot for cumulative time replication at the 1000-m endpoint. The y-axis represents achieved minus target time. Solid and dashed horizontal lines denote mean bias and the 95% limits of agreement, respectively.

Supplementary Figure S5. Bland–Altman plot for mean power replication at the 1000-m endpoint. The y-axis represents achieved minus target power. Solid and dashed horizontal lines denote mean bias and the 95% limits of agreement, respectively.

Supplementary Figure S6. Bland–Altman plot for mean stroke-rate replication at the 1000-m endpoint. The y-axis represents achieved minus target stroke rate. Solid and dashed horizontal lines denote mean bias and the 95% limits of agreement, respectively.

Supplementary Figure S7. Bland–Altman plot for cumulative time replication at the 1500-m endpoint. The y-axis represents achieved minus target time. Solid and dashed horizontal lines denote mean bias and the 95% limits of agreement, respectively.

Supplementary Figure S8. Bland–Altman plot for mean power replication at the 1500-m endpoint. The y-axis represents achieved minus target power. Solid and dashed horizontal lines denote mean bias and the 95% limits of agreement, respectively.

Supplementary Figure S9. Bland–Altman plot for mean stroke-rate replication at the 1500-m endpoint. The y-axis represents achieved minus target stroke rate. Solid and dashed horizontal lines denote mean bias and the 95% limits of agreement, respectively.

Supplementary Figure S10. Participant-level cumulative endpoint trajectories for the indirectly estimated phosphagen contribution. Thin trajectories represent individual rowers and the thicker trajectory represents the group mean. Values are cumulative endpoint estimates.

Supplementary Figure S11. Participant-level cumulative endpoint trajectories for the indirectly estimated glycolytic contribution. Thin trajectories represent individual rowers and the thicker trajectory represents the group mean. Values are cumulative endpoint estimates.

Supplementary Figure S12. Participant-level cumulative endpoint trajectories for the indirectly estimated aerobic contribution. Thin trajectories represent individual rowers and the thicker trajectory represents the group mean. Values are cumulative endpoint estimates.
